# Factors Associated with Increased Survival after Surgical Resection of Glioblastoma in Octogenarians

**DOI:** 10.1371/journal.pone.0127202

**Published:** 2015-05-15

**Authors:** Kalil G. Abdullah, Ashwin Ramayya, Jayesh P. Thawani, Lukasz Macyszyn, Maria Martinez-Lage, Donald M. O’Rourke, Steven Brem

**Affiliations:** 1 Department of Neurosurgery, Hospital of the University of Pennsylvania, Perelman School of Medicine, University of Pennsylvania, Philadelphia, Pennsylvania, United States of America; 2 Department of Pathology and Laboratory Medicine, Hospital of the University of Pennsylvania, Perelman School of Medicine, University of Pennsylvania, Philadelphia, Pennsylvania, United States of America; University of Michigan School of Medicine, UNITED STATES

## Abstract

Elderly patients with glioblastoma represent a clinical challenge for neurosurgeons and oncologists. The data available on outcomes of patients greater than 80 undergoing resection is limited. In this study, factors linked to increased survival in patients over the age of 80 were analyzed. A retrospective chart review of all patients over the age of 80 with a new diagnosis of glioblastoma and who underwent surgical resection with intent for maximal resection were examined. Patients who had only stereotactic biopsies were excluded. Immunohistochemical expression of oncogenic drivers (p53, EGFR, IDH-1) and a marker of cell proliferation (Ki-67 index) performed upon routine neuropathological examination were recorded. Stepwise logistic regression and Kaplan Meier survival curves were plotted to determine correlations to overall survival. Fifty-eight patients fit inclusion criteria with a mean age of 83 (range 80–93 years). The overall median survival was 4.2 months. There was a statistically significant correlation between Karnofsky Performance Status (KPS) and overall survival (P < 0.05). There was a significantly longer survival among patients undergoing either radiation alone or radiation and chemotherapy compared to those who underwent no postoperative adjuvant therapy (p < 0.05). There was also an association between overall survival and lack of p53 expression (p < 0.001) and lack of EGFR expression (p <0.05). In this very elderly population, overall survival advantage was conferred to those with higher preoperative KPS, postoperative adjuvant therapy, and lack of protein expression of EGFR and p53. These findings may be useful in clinical decision analysis for management of patients with glioblastoma who are octogenarians, and also validate the critical role of EGFR and p53 expression in oncogenesis, particularly with advancing age.

## Introduction

The clinical paradigm for the treatment of elderly patients with glioblastoma (GBM) remains controversial [1–5]. Among all patients, the median survival time after GBM diagnosis remains 15 months with aggressive surgical resection, chemotherapy, and radiation [6]. Elderly patients often have more medical comorbidities and lower Karnofsky Performance Scales (KPS) than their younger counterparts. This has led to an increased focus on utility and efficacy of surgical and medical treatment in geriatric patients, with some studies showing increases in life expectancy with aggressive management [7–12], possibly correlated to a higher extent of resection [13,14]. What is even less clear is the role of these therapies in patients over the age of 80, as most studies that evaluate a “geriatric” population report on patients aged 65–80. In this study, we present what is, to our knowledge, the largest cohort of patients in their 8^th^ decade and older who have undergone surgical resection for glioblastoma and factors associated with prolonged survival.

## Methods

After approval from the University of Pennsylvania Institutional Review Board, a retrospective review was conducted of all patients who presented to the Hospital of the University of Pennsylvania over the age of 80 with a new diagnosis of glioblastoma between January 1^st^, 2003 and December 31^st^, 2013 and underwent surgical resection. All patients presenting as an index glioblastoma resection over the age of 80 were included. Only secondary resections and patients undergoing biopsy alone were excluded. Patient demographics, including age, sex, race, presenting symptoms, pre and postoperative neurological examinations, KPS, intraoperative and postoperative complications, tumor location, use of adjuvant chemotherapy and radiation, immunohistochemical staining of p53 (positive vs negative), epidermal growth factor receptor (EGFR, positive versus negative), and mutated isocitrate dehydrogenase 1 (IDH1, R132H (present versus absent), time to progression, and overall survival time were recorded. Gross total resection was defined as imaging findings consistent with “no residual tumor”, and all other imaging findings were considered sub-total resections. The Karnofsky Performance Status is a validated index that allows for patient classification based on their degree of functional impairment [15]. A higher KPS is representative of better functioning (e.g. a score of 100 is considered to be a patient without evidence of any impairment, a score of 10 is a moribund patient). KPS was assigned retrospectively from the history and physical found in the medical record of each patient. Multiple stepwise logistic regression was used to perform multivariate analysis, and Kaplan-Meier survival curves were used to evaluate survival times among groups. Two-tailed T-tests and Wilcoxon-Rank Sum tests as well as multiple analysis of variance was used to analyze differences between groups. The P-value was set at 0.05. All statistics were performed using JMP 11.2.1 (SAS Institute, Cary, North Carolina, USA).

## Results

Fifty-eight patients with a mean age of 83 (range 80–93) years fit inclusion criteria (Table 1). The most common comorbidities were hypertension (76%), hyperlipidemia (48%), and coronary artery disease (36%). The majority of patients had a preoperative KPS of 70 (35%) or 80 (53%), together accounting for 88% of all study patients. The median time to diagnosis was one month from the most common presenting symptom, which was an alteration in mental status (55%, Table 2). Most tumors were confined to the frontoparietal lobes (69%).

**Table 1:** Demographics.

*Characteristic*	*N = 58*	*P-value*
Age, years	83 (80–93)	NS
Male	23 (40%)	NS
***Race***		NS
African Descent	8 (14%)	
Caucasian	43 (74%)	
Other	7 (12%)	
***Comorbidities***		NS
Diabetes	15 (26%)	
Hypertension	44 (76%)	
Coronary artery disease	21 (36%)	
Hyperlipidemia	28 (48%)	
History of cancer	13 (22%)	
PE/DVT	14 (23%)	
***KPS***		*P* < 0.05
60	3 (5%)	
70	20 (35%)	
80	31 (53%)	
90	4 (7%)	

Legend: Legend: Values provided as mean (with range) or absolute number with percentage. Student’s t-tests were utilized to analyze the differences between basic demographics, and multiple analysis of variance (ANOVA) was used to analyze the differences between multiple groups. PE = pulmonary embolism. DVT = deep vein thrombosis. KPS = Karnofsky Performance Scale

**Table 2:** Presenting symptoms and tumor characteristics.

*N = 58*	
***Presenting Symptom***	
Seizure	6 (10%)
Headache	17 (28%)
Altered mental status	34 (55%)
Cranial nerve palsy	12 (7%)
Sensorimotor deficit	20 (33%)
Aphasia	16 (27%)
***Tumor characteristics***	
Multifocal	7 (12%)
Eloquent	16 (27%)
Frontoparietal	42 (69%)
Occipital	7 (12%)
Brainstem involvement	1 (2%)
Collosal	5 (9%)

**Fig 1:**
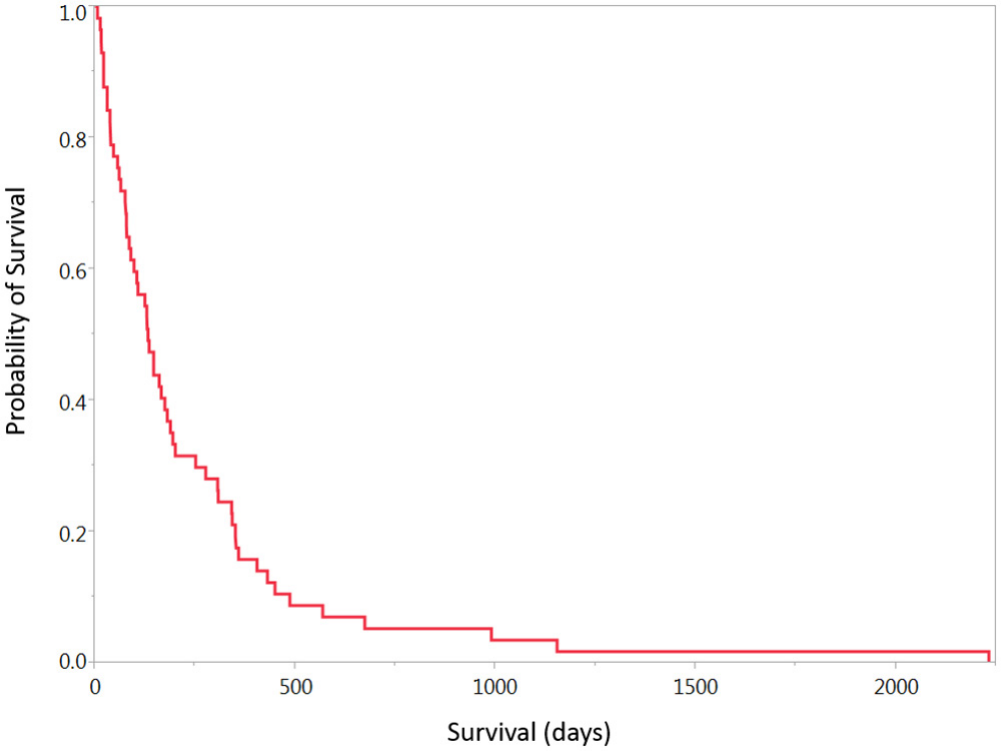
Overall survival curve.

**Fig 2:**
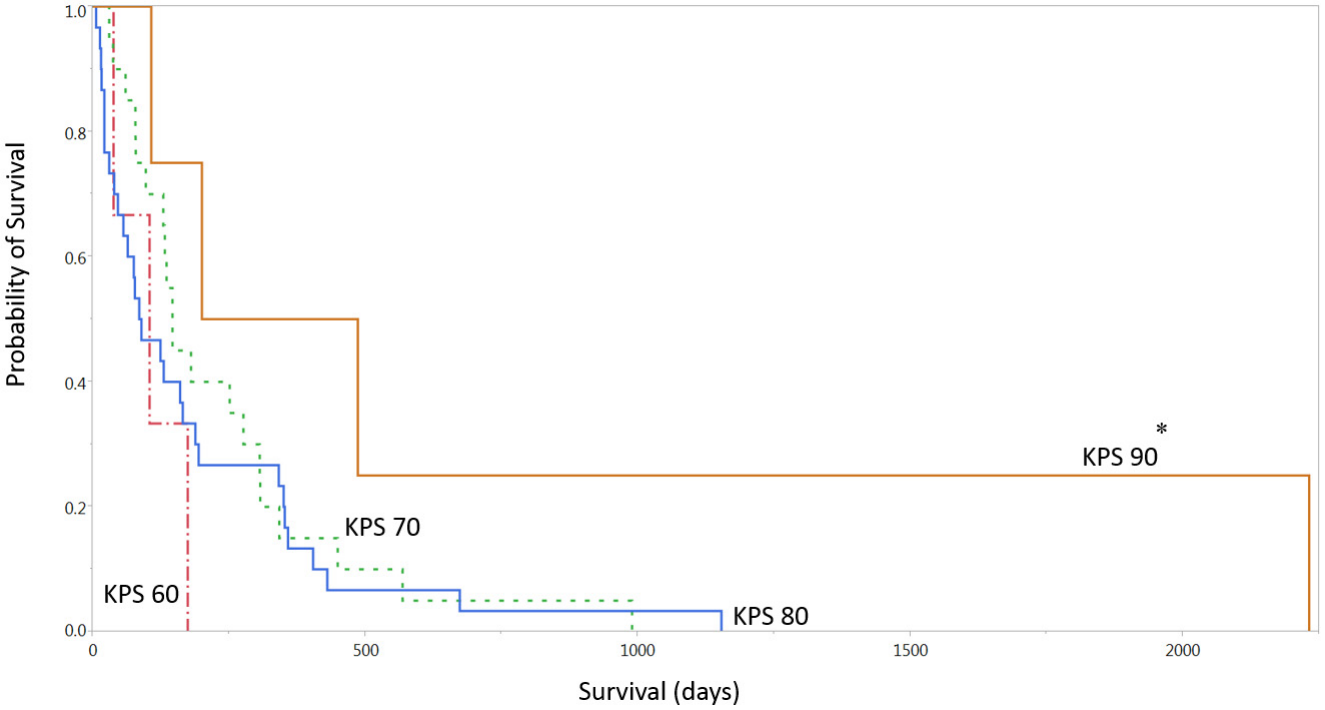
Survival curve by KPS. Legend: KPS = Karnofsky Performance Scale. * Denotes p < 0.05.

**Fig 3:**
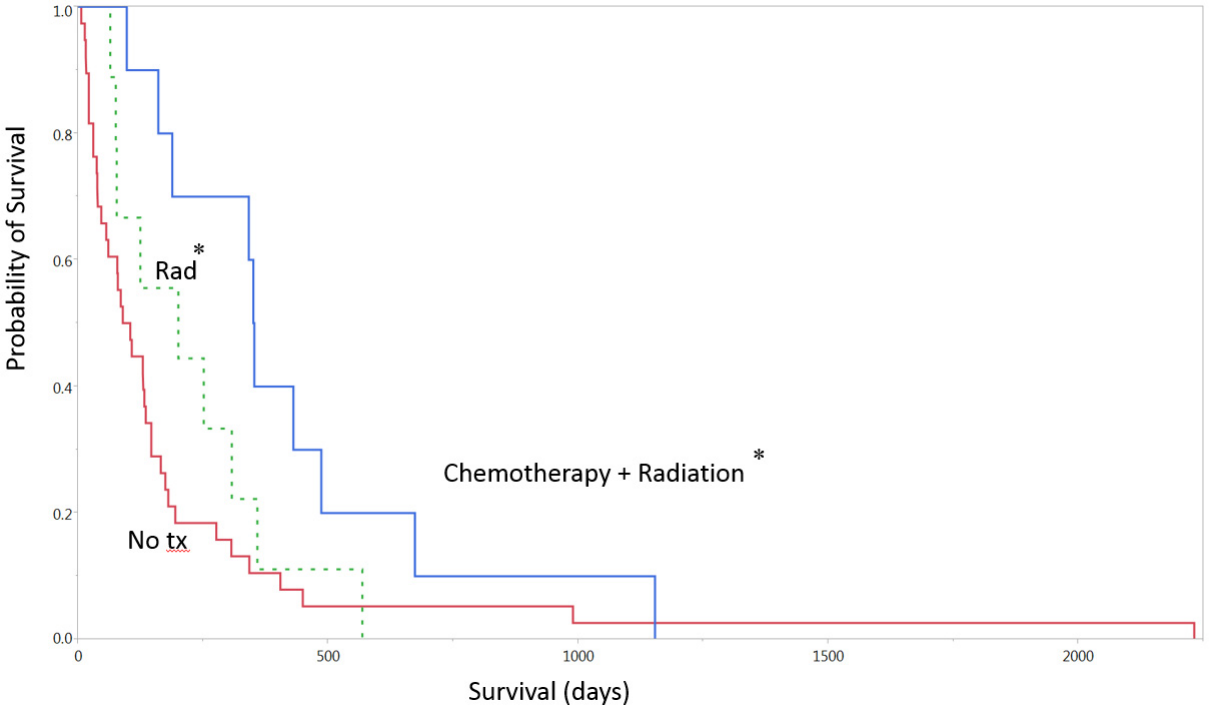
Survival curve by adjuvant therapy. * Denotes p < 0.05. Tx = treatment. Rad = radiation.

The overall median survival (OS) was 4.2 months (0.2–72 months, Fig 1). There was a statistically significant association between a higher KPS and overall survival (P < 0.05, Table 1, Figs 1 and 2). There was no association between age, race, sex, comorbidities, or radiographic tumor characteristics and overall survival. There was no association between KPS and EGFR, Ki-index, IDH1 or p53 status (p >0.05). Gross total resection was achieved in 21% of patients, with no significant outcome on survival (Table 3). The rate of overall postoperative thrombotic event was 10%, and the 30-day readmission rate was 17%. The median intensive care unit length of stay was 1 day and the median overall hospital stay was 6 days, neither was associated with OS. The most common disposition was inpatient neurorehabilitation (53%). The majority of patients did not undergo any adjuvant therapy (66%), with 10 undergoing radiation alone, and 10 undergoing radiation and chemotherapy (17%). There was a significant difference in survival among these three groups, with a median survival of 96.5, 200, and 351 days for patients with no adjuvant therapy, radiation alone, and chemoradiation (P <0.05. Fig 3).

**Table 3:** Postoperative Profiles.

*Characteristics*		*P-value*
***Resection Status***		
Gross total resection	12 (21%)	NS
Subtotal Resection	40 (69%)	
***Postoperative Complications***		
Surgical site infection	1 (2%)	
Seizure	5 (9%)	
Deep vein thrombosis	5 (9%)	
Pulmonary embolism	1 (2%)	
30-day readmission	10 (17%)	
***Adjuvant treatment***		
No adjuvant therapy	38 (66%)	
Radiation only	10 (17%)	*P* < 0.05
Radiation + chemotherapy	10 (17%)	
***Disposition***		
ICU length of stay	1 (1–30 days)	NS
Hospital length of stay	6 (2–30 days)	NS
Home	14 (24%)	
Rehab	31 (53%)	
SNF	9 (16%)	
Hospice	2 (3%)	
Inpatient death	2 (3%)	

Legend: Values provided as median (with range) or absolute numbers with percentage. Wilcoxon Rank Sum tests were used to determine differences in overall survival between those who underwent gross total and subtotal resection, as well as survival differences in the adjuvant treatment group and length of stay parameters. NS = not significant (p > 0.05). ICU = intensive care unit. SNF = skilled nursing facility.

**Fig 4:**
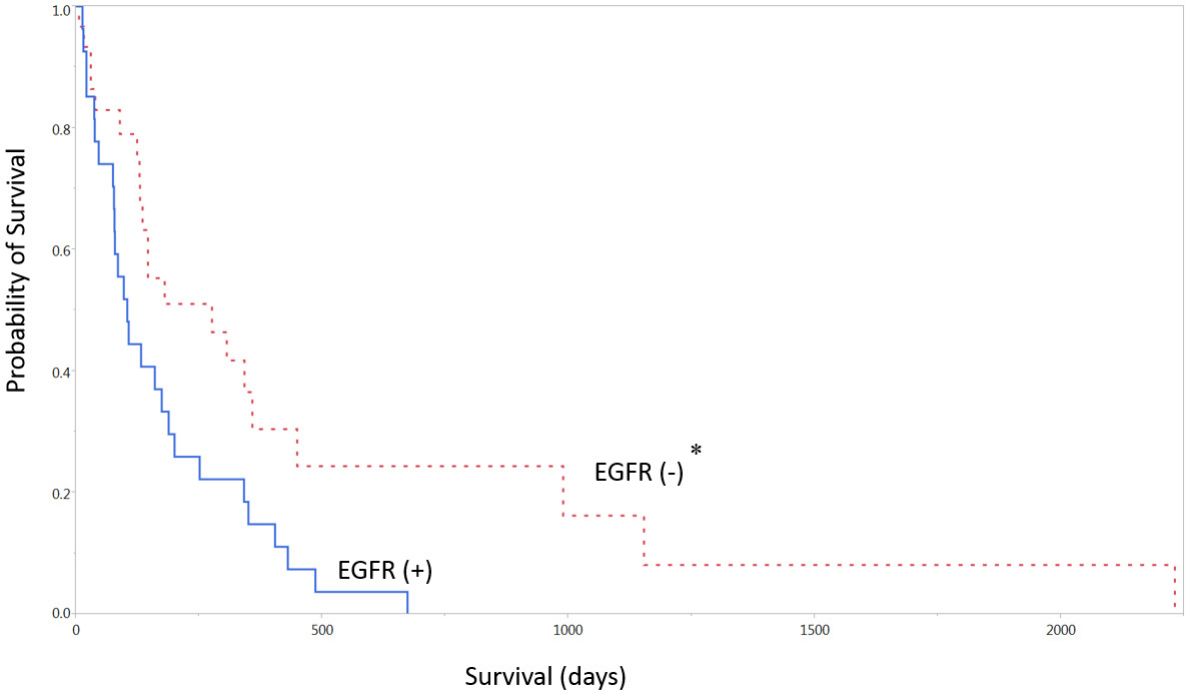
EGFR expression and survival. EGFR = epidermal growth factor receptor. * Denotes p < 0.05.

**Fig 5:**
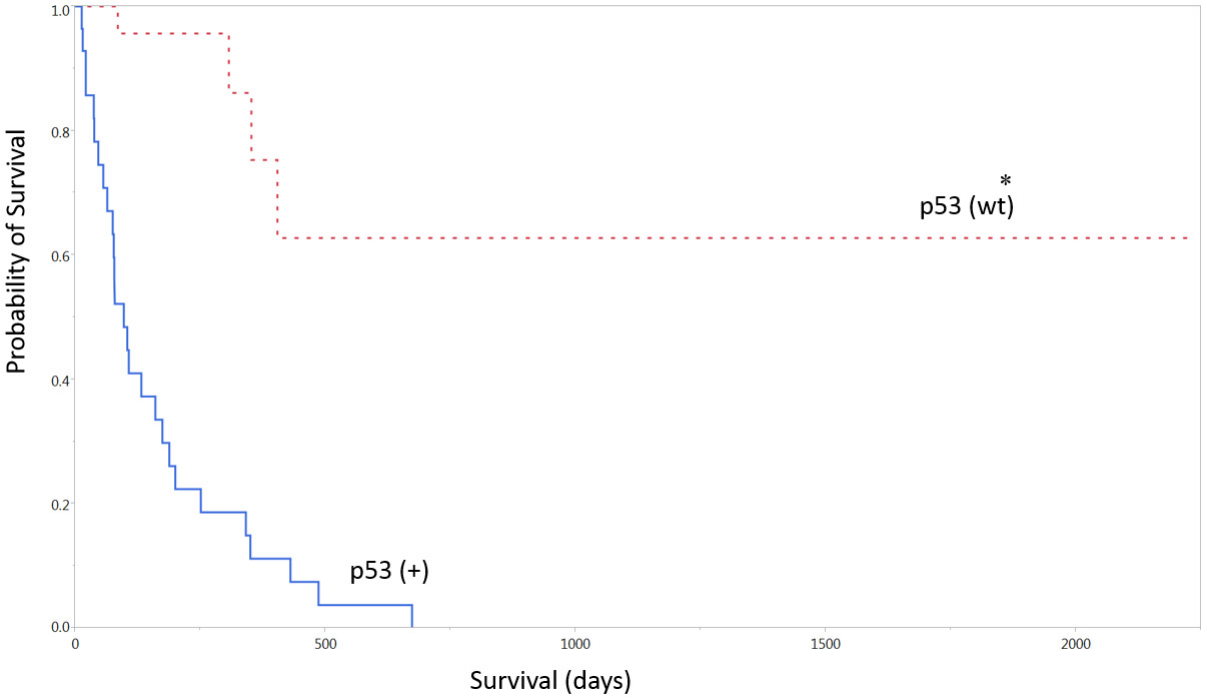
P53 expression and overall survival. Wt = wild type. * Denotes p < 0.05.

There was a statistically significant association between lack of EGFR immunohistochemical expression and increased survival (p < 0.05, median survival 276 vs. 104 days, Table 4, Fig 4). There was also an association between lack of p53 immunohistochemical expression and increased survival (p < 0.001, median survival 97 vs. 330, Fig 5). There was no statistical correlation between p53 and EGFR expression, or combined survival benefit between lack of both p53 and EGFR expression (p > 0.05). There were no cases of mutated IDH-1 in this cohort (0/22) in those patients with available IDH-1 staining. There was no association between the Ki-67 proliferation index and overall survival.

**Table 4:** Immunohistochemical staining.

*Protein*		*P-value*
EGFR (+)	20 (35%)	
EGFR (-)	27 (47%)	P < 0.05
p53 (+)	27 (47%)	
p53 (wt)	4 (14%)	P < 0.05
Mutant IDH1 (+)	0 (0%)	
Mutant IDH (-)	22 (38%)	
Ki index (< 30%)	26 (77%)	NS
Ki index (31 to 50%)	5 (15%)	
Ki index (>50%)	3 (9%)	

Legend: EGFR = epidermal growth factor receptor. IDH1 = isocitrate dehydrogenase. Ki index = Antigen Ki-67 index. EGFR (+) = positive staining for the epidermal growth factor receptor. EGFR (-) = no staining for the epidermal growth factor receptor was present. P53 (+) = presence of p53 immunohistochemical staining. P53 (wt)- wt = wild type. No immunohistochemical p53 staining was found. Total simple size for EGFR was 47 patients. Total simple size for P53 staining was 31. IDH1 staining was available in 22 patients. Ki index was available in 34 patients.

## Discussion

The decision to proceed with surgical resection of glioblastoma in an elderly patient requires an informed understanding of potential outcomes and perioperative morbidity. This can be particularly challenging in patients who are substantially older than the patient population that is most commonly reported upon. Often times, “geriatric” patients are referred to as age 65 or 70 “onwards”. However, the practical and clinical features of patients in their late 6^th^ decade are most likely very different than those of patients in their late 8^th^ decade. Even in the light of recent studies that reveal that both surgical resection (versus biopsy) and aggressive treatment with chemoradiation may prolong life in a geriatric population, the decision to proceed with glioblastoma resection in an octogenarian can be particularly complex. In this study, we examined outcomes of surgical resection of 58 patients aged 80 and older (range 80–93 years). This cohort of patients is larger and better characterized than the subset of patients in larger studies who examine geriatric patients with glioblastoma but define them as aged “65 and over” and allows for a better understanding of factors associated with outcomes after surgical resection in a very unique population. Additionally, each of the 58 patients underwent “maximal safe resection” with the intent to remove as much tumor burden as possible without resultant neurological deficit.

In this population, the majority of patients had at least one medical comorbidity as shown by the greater than 35% of patients with coronary artery disease and hyperlipidemia, and the 76% with hypertension. These factors, along with age itself, were not associated with a decrease in overall survival. This is likely a reflection of the inherent homogeneity of a patient population that has already reached its 8^th^ decade of life, at which time medical comorbidities are very common and differences in age are less important than their Karnofsky Performance Scale, consistent with several studies in the elderly GBM population [16,17]. Also consistent with this literature, KPS was significantly correlated to overall survival [18](Fig 2).

Notably in this study, the expression of the proteins EGFR and p53 had a significant impact on survival time. Patients who did not have immunohistochemical expression of EGFR and p53 had a significantly longer median survival time than those with positive expression. In this study, lack of p53 and EGFR expression were found to be independent predictors of survival. Lack of co-expression of p53 and EGFR did not confer additional benefit, and expression of EGFR and p53 were not associated with KPS. The role of p53 in glioma progression is an ongoing discussion, with a working hypothesis that the over-expression of mutated p53 may mark a more aggressive tumor biology and that TP53 mutations may participate in progression from low-grade to a higher grade lesion [19]. EGFR alteration has similarly been implicated, along with p53, in portending worse overall outcome among patients with primary GBM [20]. The findings in this study are consistent with previous literature, but also provide a possible prognostic factor in this specific age group. Another potential hypothesis, albeit preliminary, is the possibility that cell senescence associated genes may be overexpressed and may phenotypically alter the characteristics of the glioblastoma, resulting in differential survival curves [21], but future work may elucidate these mechanisms.

Several other groups have reported that temozolomide and radiation may prolong survival after glioblastoma resection [7,9,11,22], while others have advocated an abbreviated course of radiation for the very elderly [23]. Our data also shows a significantly increased survival among patients who underwent either radiation or chemoradiotherapy compared to those with no postoperative treatment. The link between the utility of adjuvant therapy and overall survival is often contingent on the ability to differentiate between preoperative survival probabilities and the benefit that adjuvant therapy may confer. This may support some previous work suggesting properly selected elderly patients may be eligible for enrollment in chemoradiotherapy trials [24].

Unlike in several other studies, we did not find extent of resection to be correlated to a higher overall survival in our cohort [7,13,14]. This is likely due to our strict definition of gross total resection as “no residual tumor” noted on post-operative imaging, but also due to our institutional bias towards “maximal safe resection” in this patient group. This implies a resection that attempts to debulk the majority of tumor burden with minimal possibility of neurological deficit.

In most populations resection of glioblastoma followed by adjuvant therapy is standard of care. In the elderly and particularly the very elderly, this becomes less clear. In this study as in others, the preoperative KPS was predictive of overall survival. Based on this, it becomes difficult to advocate for resection in patients with a KPS of 60. Conversely it is easier to support resection for patients with a KPS of 90. It is in patients with an intermediate KPS of 70 or 80 in which the other prognostic factors in this study become useful, including EGFR and p53 status. The ability to recognize the expression of these proteins as independent variables may provide some insight into the decision to be more or less aggressive with adjuvant therapy in this population.

There are multiple limitations of this study’s generalizability. First, it is a retrospective cohort of a very specific patient population and although the statistical findings were significant it lacks a *prori* power analysis necessary to be more definitive in its conclusions. Secondly, p53 and EGFR status in our institution only become standard pathology screening during 2009, and so data is limited to a shorter time period than the total enrollment period of the study. Despite these limitations, we found our conclusions well supported by our available data.

Perhaps most importantly, and not addressed in this study (or most others), are quality of life indices and their effects on patient perceptions of what is still currently a terminal diagnosis, even with maximal care. In one study, it was estimated that patients older than 75 spent more than ¾ of their remaining life in hospitals [25]. Understanding quality of life after resection and providing a maximal safe resection are important components of a surgeon’s armamentarium. In this study, we provide two proteins whose expression is associated with increased survival in a very elderly population. Further studies are necessary to better elucidate a treatment paradigm for this very specific but challenging patient population.
